# Interspecific Differences between *D. pulex* and *D. magna* in Tolerance to Cyanobacteria with Protease Inhibitors

**DOI:** 10.1371/journal.pone.0062658

**Published:** 2013-05-01

**Authors:** Christian J. Kuster, Eric Von Elert

**Affiliations:** Zoological Institute, Aquatic Chemical Ecology, University of Cologne, Cologne, Germany; Federal University of Rio de Janeiro, Brazil

## Abstract

It is known that cyanobacteria negatively affect herbivores due to their production of toxins such as protease inhibitors. In the present study we investigated potential interspecific differences between two major herbivores, *Daphnia magna* and *Daphnia pulex*, in terms of their tolerance to cyanobacteria with protease inhibitors. Seven clones each of *D. magna* and of *D. pulex* were isolated from different habitats in Europe and North America. To test for interspecific differences in the daphnids’ tolerance to cyanobacteria, their somatic and population growth rates were determined for each *D. magna* and *D. pulex* clone after exposure to varying concentrations of two *Microcystis aeruginosa* strains. The *M. aeruginosa* strains NIVA and PCC^−^ contained either chymotrypsin or trypsin inhibitors, but no microcystins. Mean somatic and population growth rates on a diet with 20% NIVA were significantly more reduced in *D. pulex* than in *D. magna*. On a diet with 10% PCC^−^, the population growth of *D. pulex* was significantly more reduced than that of *D. magna*. This indicates that *D. magna* is more tolerant to cyanobacteria with protease inhibitors than *D. pulex*. The reduction of growth rates was possibly caused by an interference of cyanobacterial inhibitors with proteases in the gut of *Daphnia*, as many other conceivable factors, which might have been able to explain the reduced growth, could be excluded as causal factors. Protease assays revealed that the sensitivities of chymotrypsins and trypsins to cyanobacterial protease inhibitors did not differ between *D. magna* and *D. pulex*. However, *D. magna* exhibited a 2.3-fold higher specific chymotrypsin activity than *D. pulex*, which explains the observed higher tolerance to cyanobacterial protease inhibitors of *D. magna.* The present study suggests that *D. magna* may control the development of cyanobacterial blooms more efficiently than *D. pulex* due to differences in their tolerance to cyanobacteria with protease inhibitors.

## Introduction

The frequency of cyanobacterial blooms in many marine and freshwater environments has increased world wide during the last century, partly due to increasing temperatures as a consequence of global warming and partly due to the eutrophication of lakes [1]. Blooms of cyanobacteria and their toxins may sometimes be associated with harmful effects on human health and livestock [2], [3]. When the temperature of the epilimnion reaches its maximum in late summer and early fall [4], the phytoplankton of many eutrophic lakes and ponds is often dominated by bloom-forming cyanobacterial species of the genera *Microcystis*, *Anabaena* and/or *Oscillatoria* [5]. During this time cyanobacteria are often an important food source for herbivorous zooplankton in freshwater ecosystems, such as for *Daphnia*, which often provides an important link for the transfer from primary production, e.g. from cyanobacteria to higher trophic levels. In cases when growth of *Daphnia* is mainly restricted by food quantity, non-toxic cyanobacteria can act as a complementary food source for *Daphnia* [6], [7]. However, since in eutrophic lakes growth of *Daphnia* is rather constrained by food quality than by food quantity, bloom-forming cyanobacteria in those habitats have been claimed to be a major factor for a constrained mass and energy transfer from primary producers to organisms of higher trophic levels [8], [9].

Negative relationships between bloom-forming cyanobacteria and the abundance of *Daphnia* have been discussed extensively over the years, and three major quality constraints of cyanobacteria as a food source have been revealed so far: (1) The occurrence of cyanobacterial filaments and the formation of colonies hinder ingestion by interfering with the filtering apparatus of *Daphnia* [10]. (2) Compared to most green algae, cyanobacteria contain low levels of essential lipids such as highly unsaturated fatty acids and sterols, which leads to reduced somatic and population growth of *Daphnia* due to constrained carbon assimilation [11]–[14]. (3) Many cyanobacteria produce a variety of bioactive secondary metabolites such as hepatotoxins like microcystins [15] and/or protease inhibitors [16]–[18]. These compounds reduce the fitness of *Daphnia* in terms of survival, growth and reproduction [19], [20]. In addition to microcystins (which are the most extensively investigated class of cyanobacterial toxins), the role of protease inhibitors in herbivore/cyanobacteria interaction has recently also become a focus of attention. More than twenty depsipeptides, which specifically inhibit the serine proteases chymotrypsin and trypsins, have been found in different genera of marine and freshwater cyanobacteria [16]. These two classes of proteases are the most important digestive enzymes in the gut of *D. magna* and are responsible for more than 80% of the proteolytic activity [21].

It is known that the edible size fraction of natural phytoplankton can contain compounds that inhibit *Daphnia*’s trypsins and chymotrypsins [22]. This inhibitory potential of seston can be in the same order of magnitude as of pure cyanobacterial cultures [23]. Hence, it is reasonable to assume that an interference of cyanobacterial protease inhibitors with *Daphnia*’s digestive proteases occurs in nature and is ecologically relevant.

However, several studies have also demonstrated that *Daphnia* may develop tolerances against cyanobacterial toxins at the population level [24]–[27]: *D. magna* populations that were pre-exposed to toxic cyanobacteria exhibited a higher tolerance to microcystin producing *M. aeruginosa* than populations that were not pre-exposed [25]. Furthermore, Sarnelle & Wilson [24] suggested that *D. pulicaria* populations, exposed to high cyanobacterial levels over long periods of time, can adapt in terms of being more tolerant to dietary toxic cyanobacteria. With regard to protease inhibitors Blom *et al.* [27] have shown that *Daphnia* sp. coexisting with *Planktothrix rubescens* (a cyanobacterium that contains the trypsin inhibitor oscillapeptin-J) was significantly more tolerant to oscillapeptin-J than *Daphnia* sp. from a lake free of this cyanobacterium. Considering the finding that almost 60% of 17 cyanobacterial blooms isolated from 14 distinct water-bodies in India contained protease inhibitors [28], it is reasonable to assume that increased tolerance to cyanobacteria in *Daphnia* populations may be caused by an enhanced tolerance to the cyanobacterial protease inhibitors. It has been suggested that at least two fundamental mechanisms underlie the increased tolerance to these dietary inhibitors: (1) Colbourne *et al.* [29] have hypothesized that the ability of *Daphnia* to cope with different environmental conditions is a consequence of an elevated rate of gene duplications resulting in tandem gene clusters. And indeed, a surprisingly high number of genes of digestive serine proteases have been found in the recently published genome of *D. pulex* [29]. (2) Von Elert *et al.* [18] have shown that a physiological plasticity at the protein level in *Daphnia* in terms of expressing different isoforms of digestive enzymes leads to increased tolerance against cyanobacterial protease inhibitors.

In the present study we tested for interspecific differences between two *Daphnia* species (*D. magna* and *D. pulex*) in their tolerance to cyanobacteria which produce protease inhibitors. *D. pulex* and *D. magna* are both large-bodied species and are frequently encountered in fishless ponds [30]. Due to the availability of full-genome data (*D. pulex*, [29]) or EST libraries (*D. magna*; [31]), both *Daphnia* species are ideal for ecological investigations and were therefore chosen for use in the present study. To determine potential differences between *D. pulex* and *D. magna* in their tolerance to cyanobacteria containing protease inhibitors, we performed single-clone somatic and population growth experiments in which the clones were fed with various cyanobacterial mixtures containing trypsin or chymotrypsin inhibitors. Both *M. aeruginosa* strains used in the present study (NIVA Cya 43 and PCC7806^−^) produce exclusively either the chemically known chymotrypsin inhibitors cyanopeptolin 954 and nostopeptin 920 (NIVA, [32]) or specific cyanopeptolins (A-D) which are known to inhibit trypsins (PCC^−^, [33]). Possible differences in tolerance to cyanobacteria with protease inhibitors might have several causes and are therefore tested in the present study: (1) We determined the specific trypsin and chymotrypsin activity of each of the investigated *D. magna* and *D. pulex* clones and hypothesized that high growth rates on cyanobacterial diets might result from high specific protease activities. (2) For each *Daphnia* clone, we determined the sensitivity of gut chymotrypsins and trypsins to the respective cyanobacterial protease inhibitors. We assumed that higher sensitivity values of *Daphnia*’s gut proteases might cause reduced somatic and population growth rates for diets with cyanobacterial protease inhibitors.

## Materials and Methods

### Origin and Cultivation of Organisms

Two cyanobacterial strains and one green alga were used in the single-clone growth experiments: The cyanobacterium *Microcystis aeruginosa* NIVA Cya 43 (Culture Collection of Algae, Norwegian Institute for Water Research), subsequently labeled as ‘NIVA’, is known to contain the chymotrypsin inhibitors cyanopeptolin 954 and nostopeptin 920 [32]. NIVA was cultured in 2 l chemostates in sterile cyano medium [34] at a dilution rate of 0.1 d^−1^ (20°C; illumination: 40 µmol m^−2^ s^−1^). *M. aeruginosa* PCC 7806 Mut contains the trypsin inhibitors cyanopeptolin A–D [33] and was grown in 0.75 l chemostates under otherwise identical conditions as for NIVA. *M. aeruginosa* PCC 7806 Mut is a genetically engineered microcystin synthetase knock-out mutant of *M. aeruginosa* PCC 7806 [35] and is subsequently labeled as ‘PCC^−^’. Neither *M. aeruginosa* strain contains microcystins. The green algae *Chlamydomonas* sp. (strain 56, culture collection of the Limnological Institute at the University of Constance) was grown in 5 l semi-continuous batch cultures (20°C; illumination: 120 µmol m^−2^ s^−1^) by replacing 20% of the culture with sterile cyano medium every Monday, Wednesday and Friday in the late exponential phase of the culture. *Chlamydomonas* sp. contains neither chymotrypsin/trypsin inhibitors nor microcystins.

Seven *D. magna* and seven *D. pulex* clones originating from different habitats (Table 1) were used in the somatic and population growth experiments. All clones were cultured separately in aged, membrane-filtered tap water and fed with saturating concentrations of *Chlamydomonas* sp. for at least three generations prior to the experiment.

**Table 1:** Geographic origin of the *Daphnia* clones used in the experiments.

Daphnia spp.	Clone	Location	Latitude	Longitude	Reference
*D. pulex*	Gerstel	Germany	N/A	N/A.	[51]
*D. pulex*	NFL3	USA	N39°54′	W84°55′	[52]
*D. pulex*	Gräf	Germany	N50°49′04′′	E10°42′02′′	[53]
*D. pulex*	Disp14	Canada	N42°13′	W83°02′	[54]
*D. pulex*	Povi113	USA	N42°45′	W85°21′	[52]
*D. pulex*	Giev08	Germany	N51°57′48′′	E7°34′38′′	Y. Reydelet^1^
*D. pulex*	TCO	USA	N43°49′48′′	W124°08′53′′	[29]
*D. magna*	F10	Germany	N50°56′02′′	E6°55′41′′	[22]
*D. magna*	G38	Belgium	N51°04′04′′	E3°46′25′′	This study
*D. magna*	S15	Sweden	N55°40′31′′	E13°32′42′′	[55]
*D. magna*	P6	Poland	N52°19′21′′	E20°43′49′′	[55]
*D. magna*	P	The Netherlands	N51°44′01′′	E5°08′17′′	[56]
*D. magna*	B	Germany	N54°19′39′′	E10°37′45′′	[57]
*D. magna*	W	Poland	N/A	N/A	[58]

1 Y. Reydelet, personal communication, 2011.

### Somatic and Population Growth Assays

Each of the 14 clones was assayed in single-clone experiments for somatic and population growth. Five (*D. magna*) to seven (*D. pulex*) juveniles from the same cohort of the third clutch and no older than 24 h were kept in 0.25 l aged and filtered tap water (membrane filter of 0.45 µm pore size) under constant dim light at 20°C. The animals were fed with non-limiting food concentrations (2 mg C/l) either of 100% *Chlamydomonas* sp. or of various mixtures of *Chlamydomonas* sp. and the two *M. aeruginosa* strains: In two treatments the animals were fed either with a mixture of 80% *C*. sp and 20% NIVA or with a mixture of 50% *Chlamydomonas* sp. and 50% NIVA. In one further treatment the animals were fed with a mixture of 90% *Chlamydomonas* sp. and 10% PCC^−^. Each treatment was triplicated, and animals were transferred daily into fresh water with saturating food concentrations. Somatic growth rates were calculated on day six as according to Wacker & Von Elert [36] as
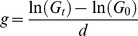
for which (G) is the body dry-weight of a subsample of the animals at the beginning (*G*_0_) and end (*G*_t_) of the experiment. Mean individual dry weights were mean values of two individuals. As according to Brzezinski & Von Elert [37] population growth rates (r) were calculated from daily survival and fecundity of the first clutch by using Euler’s equation,



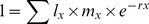
in which *l_x_* is the survival rate and *m_x_* the size of the first clutch on day *x*. Population and somatic growth rates were calculated for each replicate and subsequently averaged to give the mean of the treatment. Population growth rates, calculated on the basis of fecundity at first reproduction, may be a better predictor of fitness in the presence of size-selective mortality than the calculation based on the first three clutches as used in Lampert & Trubetskova [38], since larger individuals are more vulnerable to visually orientated predators. We performed an equal variance test (Levene’s test) for each treatment to ensure a comparable intraspecific variability of both *Daphnia* species.

### Preparation of Cyanobacterial Extracts and *Daphnia* Homogenates

Freeze-dried NIVA or PCC^−^ were thoroughly homogenized and mixed separately. 50 mg of the resulting powder was suspended in 500 µl of 60% methanol and sonicated for 15 min followed by centrifugation (3 min at 10^4^×g). The supernatant was subsequently separated from the residue and used as an extract in the protease assays. Controls with 60% methanol had no effects on protease activities.

Seven-day-old individuals of each of the seven *D. magna* and *D. pulex* clones grown on non-limiting food concentrations of *Chlamydomonas* sp. were homogenized with a Teflon pestle. Subsequently the homogenate was centrifuged (3 min at 1.4×10^4^×g). In order to minimize protease inactivation due to autolytic degradation, the resulting supernatant was kept permanently on ice until it was used in the enzyme assays.

### Protease Activity and Protease Inhibition Assays

The activity of the proteases chymotrypsin and trypsin in homogenates of all *Daphnia* clones was measured as according to Von Elert *et al.* [21]. The protein concentration of the supernatant was analyzed using a Qubit fluorometer and the appropriate Quant-iT™ Protein Assay Kit (Invitrogen, Carlsbad, USA) as according to the manufacturer’s standard protocol. For the protease activity and protease inhibition assays, SuccpNA (N-succinyl-L-alanyl-L-alanyl-L-prolyl-Lphenylalanine 4-nitroanilide, Sigma, 125 µM in DMSO) was used as a substrate for chymotrypsins, while BapNA (N-R-benzoyl-DL-arginine 4-nitroanilide hydrochloride, Sigma, 1.8 mM in DMSO) served as a substrate for trypsins. Trypsin and chymotrypsin assays were performed in a potassium phosphate buffer (0.1 M, pH 7.5). The absorption change was measured continuously for 10 min at 30°C at 390 nm with a Cary 50 photometer (Varian). Specific proteolytic activity was determined as nmol *para*-nitroanilide liberated per minute and µg protein for synthetic substrates. With regard to the chymotrypsin inhibition assays, homogenates of each of the *D. magna* and *D. pulex* clones were assayed after addition of 10–15 different concentrations of the NIVA extract. The resulting chymotrypsin activities were plotted as a function of extracted NIVA biomass per ml assay volume. By fitting a sigmoidal dose response curve, the concentration of extracted NIVA biomass which resulted in a 50% inhibition of *Daphnia* chymotrypsin activity (IC_50_) was calculated. The higher the IC_50_ values for the analyzed *D. magna* and *D. pulex* homogenates, the more tolerant their respective chymotrypsins were to chymotrypsin inhibitors from NIVA. Trypsin inhibition of the *D. magna* and *D. pulex* clones was assayed as above, except that different concentrations of the PCC^−^ extract were used.

### Data Analysis

For the inhibition assays of each single clone of *D. magna* and *D. pulex*, the protease activities were plotted as a function of extracted NIVA or PCC^−^ biomass. Resulting IC_50_ values were calculated by fitting a sigmoid dose-response curve using the software Graph Pad Prism (GraphPad Software, Inc.). A Mann-Whitney U-test was used to determine whether one of the two *Daphnia* species exhibits stronger growth reductions on mixtures of *M. aeruginosa*, while a Student’s t-test was used to determine interspecific differences in protease inhibition and specific protease activity assays between the two species. Population growth rates were calculated for each individual per replicate independently using an R-script and subsequently averaged for each replicate. Mean Population growth rate of a *Daphnia* clone on a diet derived from the average of three replicates. All other statistical tests were performed using SigmaPlot 11 (Systat Software, Inc.). A significance level of p = 0.05 was applied to all statistical analyses.

## Results

**Figure 1:**
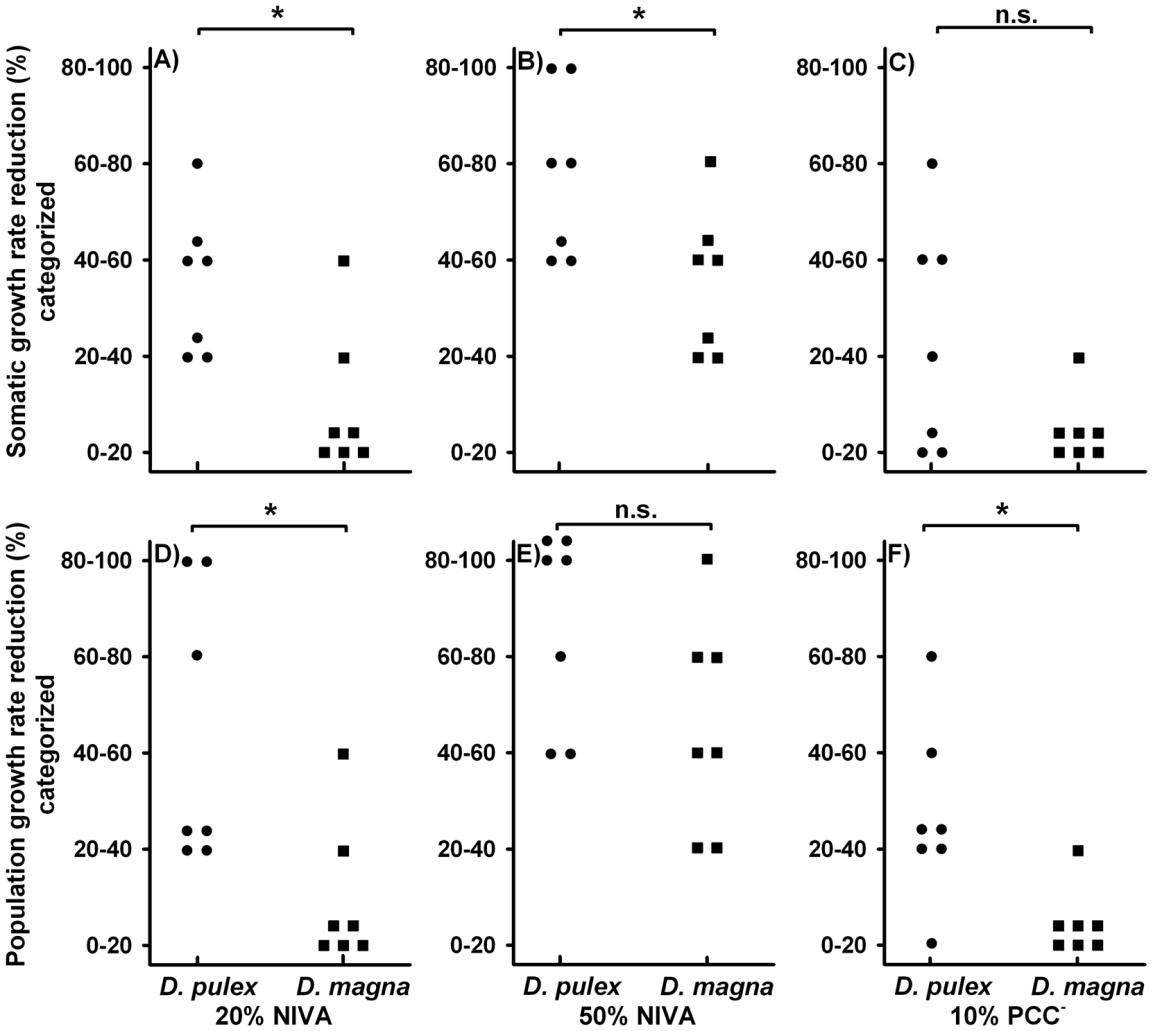
Reduction of growth rates of each *Daphnia* clone fed on NIVA and PCC^−^. Reduction of relative somatic (a, b, c) and population (c, d, e) growth rates of clones of *D. pulex* (circles) and *D. magna* (squares) in response to different mixtures of *Chlamydomonas* sp. and either of two *Microcystis aeruginosa* strains, NIVA or PCC^−^. Animals were fed either with 80% *Chlamydomonas* sp. and 20% NIVA (a, d), with 50% *Chlamydomonas* sp. and 50% NIVA (b, e) or with 90% *Chlamydomonas* sp. and 10% PCC^−^ (c, f). *Daphnia* clones were classified into growth reduction categories according to the relative growth rate reduction on respective treatments compared to the control treatment on 100% *Chlamydomonas* sp. Significant differences (Mann-Whitney U-test, p<0.05) between species are indicated by an asterisk, while no differences are labeled with “n.s.”.

In order to test for interspecific differences between *D. pulex* and *D. magna* with regard to their tolerance to cyanobacteria, we performed population and somatic growth experiments on four different diets. Seven clones of each species, each originating from different habitats, were fed either with pure *Chlamydomonas* sp. or with mixtures of *Chlamydomonas* sp. and either of two *M. aeruginosa* strains, NIVA and PCC^−^. For each treatment (20% NIVA, 50% NIVA and 10% PCC^−^) as well as for both growth rate calculations (somatic growth and population growth) all *D. magna* and *D. pulex* clones were categorized into five groups according to their respective relative growth rate reduction (Figure 1a–f), whereas values ≤0 were regarded as no growth rate reduction on respective diet and were consequently grouped in the category 0–20%. Raw data underlying this classification are presented in Table 2. Equal variance tests revealed that the variability for both *Daphnia* species were not different for each treatment (Levene’s test, p>0.05). On a mixture of 80% *Chlamydomonas* sp. and 20% NIVA the categorized growth rates of the *D. pulex* clones were significantly more reduced than those of the *D. magna* clones (Figure 1a, d). This applies equally for the reduction of population growth rates (*U*-test: U = 6, p<0.05) and of somatic growth rates (*U*-test: U = 6, p<0.05): While five of seven *D. magna* clones were grouped into the first category (0%–20%) of relative somatic and population growth rate reduction, no *D. pulex* clones were grouped in this category when grown on a mixture with 20% NIVA. With increasing NIVA concentration from 20% to 50%, the categorized somatic and population growth rates decreased for both *Daphnia* species (Figure 1b, e). On 50% NIVA, the somatic growth rates for *D. pulex* were significantly more reduced than for *D. magna* (Figure 1b, *U*-test: U = 8.5, p<0.05), while this was not the case for the population growth rates (Figure 1e, *U*-test: U = 12, p = 0.128). However, with regard to the population growth rate reduction on 50% NIVA, four of seven *D. pulex* clones were grouped in the category of strongest growth reduction, whereas only one clone of *D. magna* was grouped in this category (Figure 1e). On 10% PCC^−^, the majority of the *D. magna* clones (six of seven) were grouped in the category of weakest somatic- and population growth rate reduction, representing a reduction of 0%–20% compared to the control treatment on 100% *Chlamydomonas* sp. (Figure 1c, f). Population growth rates of the *D. pulex* clones exhibited a more variable classification when grown on 10% PCC^−^ and were significantly more reduced than those of the *D. magna* clones (Figure 1f, *U*-test: U = 6, p = 0.017). With regard to the somatic growth rate reduction on 10% PCC^−^ no statistical differences were found between *D. pulex* and *D. magna* (Figure 1c, *U*-test: U = 12.5, p = 0.128).

**Table 2:** Relative reduction of somatic and population growth rate of all *D. magna* and *D. pulex* clones on mixtures containing 20% NIVA, 50% NIVA and 10% PCC^−^ as well as specific activity and IC_50_ values of *Daphnia*’s chymotrypsins (CT) and trypsins (T).

		Somatic growth rate reduction (%)			Population growth rate reduction (%)			Specific protease activity		IC_50_ values	
		on			on			(nmol/min*µg prot)		(ng/ml)	
*Daphnia* spp.	Clone	20% NIVA	50% NIVA	10% PCC^−^	20% NIVA	50% NIVA	10% PCC^−^	CT	T	CT	T
*D. pulex*	Gerstel	27.7	48.0	6.6	89.2	98.7	25.1	306.43	39.7	230.7	540.8
*D. pulex*	NFL3	42.6	84.6	64.9	39.4	59.4	37.6	95.18	30.2	417.5	577.6
*D. pulex*	Gräf	59.1	57.6	7.7	92.6	100.0	30.1	228.88	67.56	311	1052
*D. pulex*	Disp14	38.6	64.0	41.2	23.7	68.9	63.4	23.45	25.4	281.1	678.6
*D. pulex*	Povi113	42.4	86.8	45.5	36.1	92.9	45.7	38.6	21.81	228.4	803.3
*D. pulex*	Giev08	73.4	59.4	0.4	79.4	100.0	17.5	116.98	66.72	296.1	944.9
*D. pulex*	TCO	27.5	63.6	20.8	32.0	57.7	25.0	222.55	64.99	304.6	994
*D. magna*	F10	52.4	76.5	17.2	46.7	80.8	19.1	353.63	51.7	195	388.5
*D. magna*	G38	7.2	50.2	−12.9	15.4	64.3	5.2	226.48	41.57	216.9	990.4
*D. magna*	S15	25.2	52.2	12.1	34.2	73.8	14.6	545.09	92.53	269.3	893.3
*D. magna*	P6	17.2	43.3	18.4	17.4	26.4	15.0	385.33	50.11	251.1	587.8
*D. magna*	P	−1.4	39.9	−9.8	−0.1	53.6	−10.9	358.1	43.24	244.2	1135
*D. magna*	B	10.9	22.4	28.9	9.8	24.3	36.6	215.75	45.23	271.1	920.3
*D. magna*	W	1.7	35.4	5.3	−43.6	53.2	−2.9	238.46	56.34	230.7	1222

**Figure 2:**
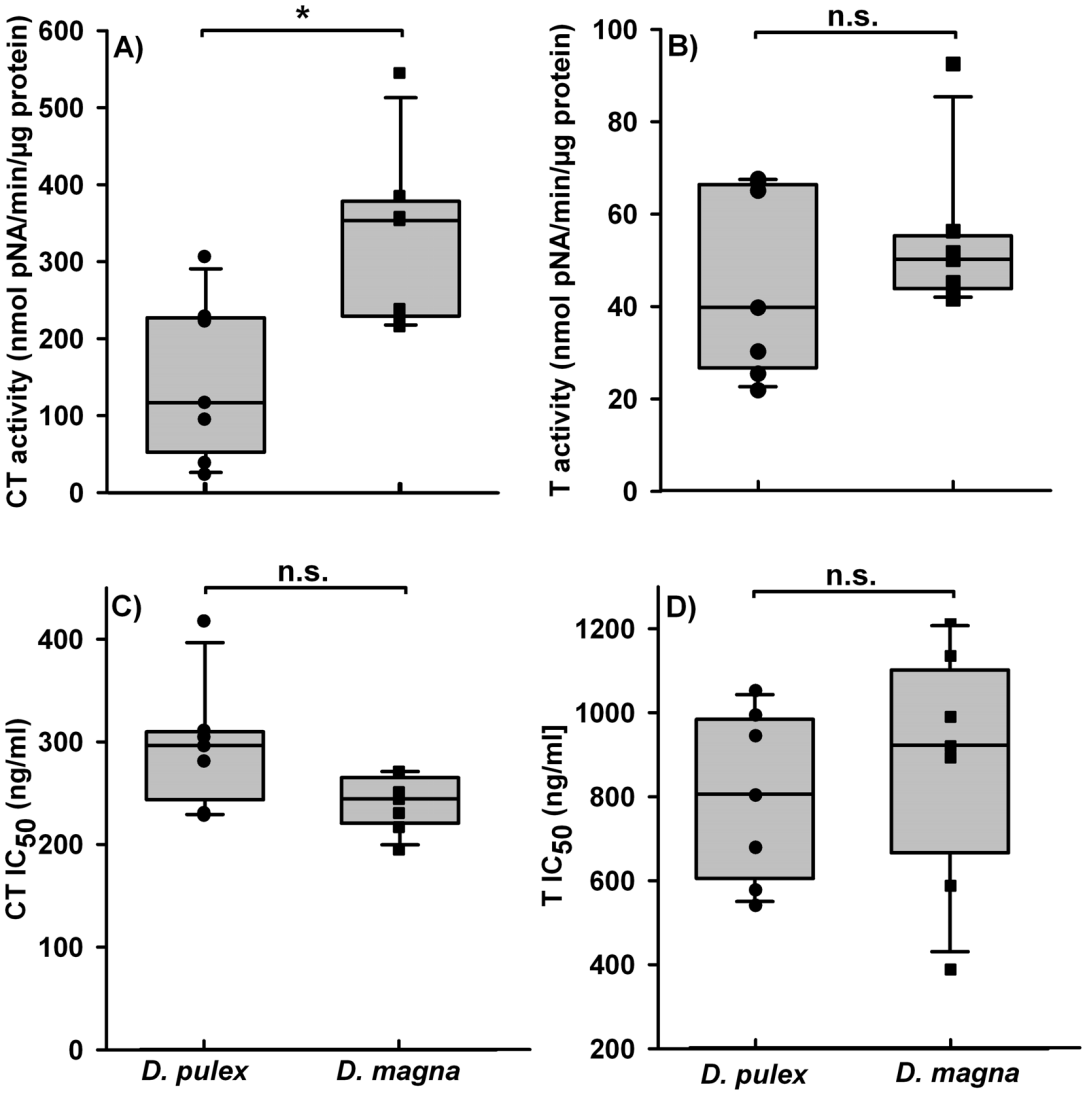
Sensitivity and specific activity of trypsins and chymotrypsins of each *Daphnia* clone. Box plots showing two possible causes for the observed differences in the tolerance of *D. magna* and *D. pulex* to cyanobacteria with protease inhibitors were tested: (a) specific chymotrypsin (CT) and (b) trypsin (T) activity of the *D. pulex* (circles) and *D. magna* clones (squares). Inhibition of digestive proteases from homogenates of clones of *D. pulex* (circles) and *D. magna* (squares): (c) effects of extracts of *M. aeruginosa* strain NIVA on chymotrypsins, and (d) effects of extracts of *M. aeruginosa* strain PCC^−^ on trypsins. Depicted are IC_50_ values, which represent the concentration of extracted dry weight of cyanobacterial biomass that is required to cause a 50% inhibition of respective proteases. Low IC_50_ values indicate a high sensitivity of proteases from the respective *Daphnia* clones. Medians are denoted by solid black lines, while the top and bottom box edges denote the first and third quartile. Whiskers denote the largest and smallest data within 1.5 times the interquartile range. Significant differences (Student’s t-test, p<0.05) among species are indicated by an asterisk; no differences are labeled with “n.s.”.

We furthermore quantified the specific activity of trypsins and chymotrypsins for each of the *D. magna* and *D. pulex* clones as possible causes for the observed differences in tolerance of the two species to cyanobacterial protease inhibitors. Mean specific chymotrypsin activity of *D. magna* (331.8±117.5 nmol/min/mg protein standard deviation (SD)) was dramatically higher (+225%) than the activity of *D. pulex* (147.4±106.8 nmol/min/mg protein SD; Figure 2a, *t*-test: t_12_ = −3.073, p<0.05). Mean specific trypsin activity for *D. pulex* was 45.2±20.6 nmol/min/mg protein SD, whereas it was 54.4±17.6 nmol/min/mg protein SD for *D. magna* (Figure 2b); however, these values were not different (*t*-test: t_12_ = −0.897, p = 0.387).

In order to test for possible additional causes of the interspecific differences in their tolerance to cyanobacterial protease inhibitors, the sensitivity of *Daphnia*’s trypsins and chymotrypsins to cyanobacterial protease inhibitors was determined. This was achieved by specifying the concentration of extracted cyanobacterial biomass that was needed to inhibit 50% (IC_50_) of either *Daphnia*’s chymotrypsins or their trypsins (Fig. 2c, d).Mean IC_50_ values of the effects of NIVA extracts on *Daphnia*’s chymotrypsins were 295.6±63.4 ng/ml SD for *D. pulex* and 239.8±27.7 ng/mL SD for *D. magna*, although these effects were not different (Figure 2c, *t*-test: t_12_ = 2.136, p = 0.054). When specifying the IC_50_ values of effects of PCC^−^ extracts on *Daphnia*’s trypsins, no differences between *D. pulex* (798.7±205.6 ng/ml SD) and *D. magna* (876.8 ng/ml ±295.2 ng/ml SD) were detected (Figure 2d, *t*-test: t_12_ = −0.574, p = 0.577).

## Discussion

Cyanobacterial blooms are harmful to many freshwater herbivorous grazers such as *Daphnia*. Owing to the dominance of cyanobacteria in eutrophic lakes and ponds in late summer [4], *Daphnia* genotypes from these habitats have to cope more frequently with the poor food quality of cyanobacterial carbon than genotypes from habitats free of cyanobacterial blooms. The causes for the poor assimilation of cyanobacterial carbon by *Daphnia* have been studied extensively in past decades, leading to the following observations: Firstly, colonial or filamentous cyanobacteria can mechanically interfere with *Daphnia*’s filtering apparatus [10]. Secondly, cyanobacteria lack essential sterols [12] and sufficient amounts of polyunsaturated fatty acids [14]. Thirdly, the production of hepaptotoxins such as microcystins can significantly reduce the fitness of *Daphnia* [19]. In the present study, all *D. pulex* clones and five out of seven *D. magna* clones exhibited a reduction in somatic and population growth rates at a concentration of 20% NIVA. The negative effects mentioned above can be ruled out as causal factors for the observed growth reductions, since the food mixtures used here consisted of saturating concentrations of the widely used, high quality reference food *Chlamydomonas* sp. Furthermore, both cyanobacterial strains as well as *Chlamydomonas* sp. were cultured as single cells and did not contain any microcystins. However, with two *D. magna* clones reductions were observed only with 50% NIVA (*D. magna* clone P and W, Table 2). This supports data of Lürling [39], who used the same strain of *M. aeruginosa* (NIVA) and reported a reduction in growth of *D. magna* clones at a concentration of ≥25% NIVA. On the mixture with PCC^−^, some *D. magna* clones and the majority of the *D. pulex* clones exhibited a reduction of population and somatic growth rate already at a concentration of 10%. Higher concentrations would probably have resulted in a significant growth rate reduction in all *D. magna* and *D. pulex* clones, since several other studies [40], [41] have reported a clear reduction in growth of daphnids at a concentration of 20% PCC^−^.

One possible explanation for the observed somatic and population growth rate reduction of the *D. magna* and *D. pulex* clones in response to cyanobacteria could be the result of dietary inhibition of either *Daphnia*’s digestive chymotrypsins or trypsins. In this study somatic and population growth rates of *D. magna* and *D. pulex* served as a measure of tolerance to microcystin-free cyanobacteria and as an approach to test for interspecific differences. In several cases it has been demonstrated that coexistence of *Daphnia* with microcystin-producing cyanobacteria leads to local adaptation of the *Daphnia* populations, as was evidenced by increased tolerance to cyanobacteria [24], [42]. These findings support the notion that the presence of cyanobacteria positively selects for more tolerant *Daphnia* genotypes.

Several studies have already demonstrated high intraspecific variability of *Daphnia*’s tolerance to cyanobacteria [24], [25]. In the present study we also found intraspecific variability of *D. magna* and *D. pulex* in the tolerance to cyanobacteria with protease inhibitors. However, an equal variance test revealed that the variability for both *Daphnia* species were not different. With regard to interspecific comparisons we have shown for the first time that *D. magna* exhibits a higher mean tolerance to cyanobacteria with protease inhibitors than *D. pulex.* Microevolutionary adaptation of *Daphnia* populations to cyanobacteria has been experimentally confirmed: Exposure of a mixed population of several *Daphnia* clones to a microcystin-producing strain of *M. aeruginosa* resulted in an enhanced tolerance in subsequent generations [25]. Such a maternally transferred increase in tolerance of *Daphnia*’s offspring generation has also been demonstrated for the microcystin-free *M. aeruginosa* strain PCC^−^ [43] which was also used in the present study.

Currently it is not clear to which extent the observed interspecific differences between *D. magna* and *D. pulex* in tolerance to cyanobacteria with protease inhibitors are affected by physiological plasticity of the clones investigated here. Using a single clone of *D. magna*, Von Elert *et al.* [18] have shown that a physiological plasticity at the protein level in *Daphnia* in terms of expressing different isoforms of digestive enzymes leads to increased tolerance to cyanobacterial protease inhibitors. In the present study we could not find a correlation between less sensitive proteases and higher tolerance to the cyanobacterial strains. The protease inhibition assays revealed that the tolerance of chymotrypsins and trypsins to cyanobacterial protease inhibitors did not differ between *D. magna* and *D. pulex*. However, the mean specific chymotrypsin activity of *D. magna* was significantly higher than that of *D. pulex*. This elevated specific chymotrypsin activity of *D. magna* coincides with less reduced somatic and population growth rates in the presence of chymotrypsin inhibitors. Schwarzenberger *et al.* [40] have demonstrated that the same *D. magna* clone as used by Von Elert *et al.* [18] responded to dietary protease inhibitors by increased expression of trypsin and chymotrypsin genes. However, it remains to be tested, if differences in plasticity of protease expression cause the observed higher tolerance to cyanobacteria with protease inhibitors in *D. magna* than in *D. pulex*. With regard to the significantly higher population growth rates of *D. magna* on PCC^−^ than those of *D. pulex*, we couldn’t find differences in species-specific trypsin characteristics, neither with respect to the specific activity nor to the respective sensitivity.

Besides protease inhibitors, the observed differences in growth on cyanobacteria between *D. magna* and *D. pulex* could also result from differences in the tolerance to other toxic compounds such as lipopolysaccharides [44] or unidentified toxins. Additionally, we cannot rule out the possibility that potentially synergistic interactions between bioactive compounds may have important negative effects on the fitness of *Daphnia*. Such synergistic effects have been reported for the toxicity of artificial nano-particles and arsenic on the fitness of daphnids [45]. However, we are not aware of any cyanobacterial metabolites, for which synergistic effects on herbivores have been demonstrated. Furthermore, when looking at possible defense mechanisms of *Daphnia* against cyanobacterial toxins, more efficient detoxification mechanisms in *D. magna* than in *D. pulex* could also be a reason for the higher tolerance of *D. magna* to the cyanobacterial diets. One possibility of reducing toxic effects in *Daphnia* is to biotransform toxins by conjugation to glutathione, which has already been demonstrated for the cyanobacterial hepatotoxin microcystin-LR [46]. However, even for this conjugation of microcystins by *Daphnia* no intra- or interspecific comparisons are available. Although similar detoxification mechanisms of protease inhibitors in *Daphnia* have not been reported so far, they cannot be excluded as a possible causal factor for the higher tolerance of *D. magna* to diets containing cyanobacteria with protease inhibitors. Additionally, Rohrlack *et al.* [47] reported a reduction of the ingestion rate in *D. galeata* after a short time exposure (15 min.) with 100% PCC^−^, which could also result in a reduction of somatic and population growth. Here, experimental individuals were fed for at least six days a PCC^−^ proportion of 10%, while 90% of the carbon resulted from the good reference food *Chlamydomonas* sp. With regard to NIVA there was no detectable inhibition of the ingestion process of one *D. magna* clone even at a proportion of 100% NIVA in the diet (Kuster, unpublished). Thus, it seems unlikely that inhibition effects of the ingestion process contributed to the observed growth reduction on cyanobacterial diets. However, since we haven’t measured ingestion rates for all *D. magna* and *D. pule*x clones used here, we could not clearly rule out this possibility.

As a result of continuing global warming, negative effects of toxic cyanobacteria on human health, livestock and the whole ecosystem will become stronger in the near future [1]. Much effort is therefore invested in preventing or controlling cyanobacterial blooms. Besides reducing nutrient input into lakes and ponds, the control of cyanobacterial blooms through biological agents such as bacteria, viruses and unicellular grazers has been discussed extensively in the last decades [48], [49]. Depending on initial conditions, *Daphnia* may also control the development of bloom-forming cyanobacteria and may even suppress established cyanobacterial blooms [50]. The present study suggests that *D. magna* may control the development of cyanobacterial blooms more efficiently than *D. pulex* due to significantly higher tolerance to cyanobacteria with protease inhibitors. In light of the increasing temperatures as a consequence of global warming, our results suggest that toxic cyanobacterial blooms and coinciding harmful effects to the ecosystem will occur more frequently in lakes with *D. pulex* than in lakes with *D. magna*.
